# The Innate Immune Response in the Marmoset during the Acute Pneumonic Disease Caused by Burkholderia pseudomallei

**DOI:** 10.1128/iai.00550-21

**Published:** 2022-03-17

**Authors:** Sarah Ngugi, Thomas Laws, Andrew J. Simpson, Michelle Nelson

**Affiliations:** a CBR division, DSTL Porton Down, Salisbury, Wiltshire, United Kingdom; b Lao-Oxford-Mahosot Hospital-Wellcome Trust Research Unit (LOMWRU), Mahosot Hospital, Vientiane, Lao People’s Democratic Republic; c Centre for Tropical Medicine and Global Health, Nuffield Department of Medicine, University of Oxford, Oxford, United Kingdom; University of Pennsylvania

**Keywords:** HLA-DR, biomarker, innate immunity

## Abstract

Burkholderia pseudomallei is the causative agent of melioidosis, a severe human infection that is difficult to treat with antibiotics and for which there is no effective vaccine. Development of novel treatments rely upon appropriately characterized animal models. The common marmoset (Callithrix jacchus) has been established at Defense Science and Technology laboratories (DSTL) as a model of melioidosis. Further analysis was performed on samples generated in these studies to provide a description of the innate immune response. Many of the immunological features described, (migration/activation of neutrophils and macrophages, activation of T cells, elevation of key cytokines IFNγ, TNF-α, IL-6, and IL-1β) have been observed in acute melioidosis human cases and correlated with prognosis. Expression of the MHCII marker (HLA-DR) on neutrophils showed potential as a diagnostic with 80% accuracy when comparing pre- and postchallenge levels in paired blood samples. Discriminant analysis of cell surface, activation markers on neutrophils combined with levels of key cytokines, differentiated between disease states from single blood samples with 78% accuracy. These key markers have utility as a prototype postexposure, presymptomatic diagnostic. Ultimately, these data further validate the use of the marmoset as a suitable model for determining efficacy of medical countermeasures against B. pseudomallei.

## INTRODUCTION

Melioidosis is a severe bacterial disease affecting thousands of people annually, with an estimated global lethality of 89,000 cases per year (1). The disease is endemic in Southeast Asia and Northern Australia and is difficult to treat, with intensive and prolonged antibiotic therapy required (1, 2).

The key features of the innate immune response to melioidosis have been studied in numerous murine studies. Research has focused on the two inbred mouse strains, the BALB/c, which is susceptible (equivalent to acute human disease), and the C57BL/6, which is relatively resistant (a model of chronic disease) (3, 4). Differences in the immune responses between these two strains have been used to define beneficial responses (5–7). By comparison, there have been relatively few studies describing the innate immune response to B. pseudomallei in a higher animal (reviewed by [8]).

Recently, the common marmoset (Callithrix jacchus), a New World nonhuman primate (NHP) species, has been developed as an infection model for experimental inhalational melioidosis (9). Marmosets offer many advantages over the use of other animal models to assess highly infectious agents including their close molecular and immunological relationship with man (10–12), their suitability for use in the development of drugs (13), their small size allowing ethical, safe housing within biocontainment restraints, as well as their low cost and availability. Acute melioidosis infections have also been reported in both the rhesus macaque and the African green monkey (14).

Previous work focused on understanding the immune profile of the naive marmoset and expanded on the range of published human cell markers with sufficient cross-reactivity to marmosets. This also served to highlight the greater similarity between marmoset and human compared to mouse and human (15). For example, the proportion of circulating neutrophils in marmoset blood is closer to the proportions found in humans, which is much higher than for mice (15, 16), an important factor considering the pivotal role of neutrophils in both acute and chronic melioidosis (7, 17–19). Also, the marmoset has been used to study the potential of an immunomodulatory phosphoantigen to stimulate Vγ9Vδ2 T cells as a treatment for inhalational melioidosis (20). This cell type is absent in mice but is expanded in human melioidosis survivors (21).

The work here uses samples generated during the development of a model of inhalational melioidosis in the marmoset (9, 22). This analysis characterizes in detail the innate immune response to inhalational melioidosis in the common marmoset in an effort to more closely model acute human disease. Sufficient detail is essential if an animal model is to provide a useful model to evaluate medical countermeasures (23). The data provided enables application of the marmoset model for this purpose.

## RESULTS

### Naïve blood cell phenotypes.

**FIG 1:**
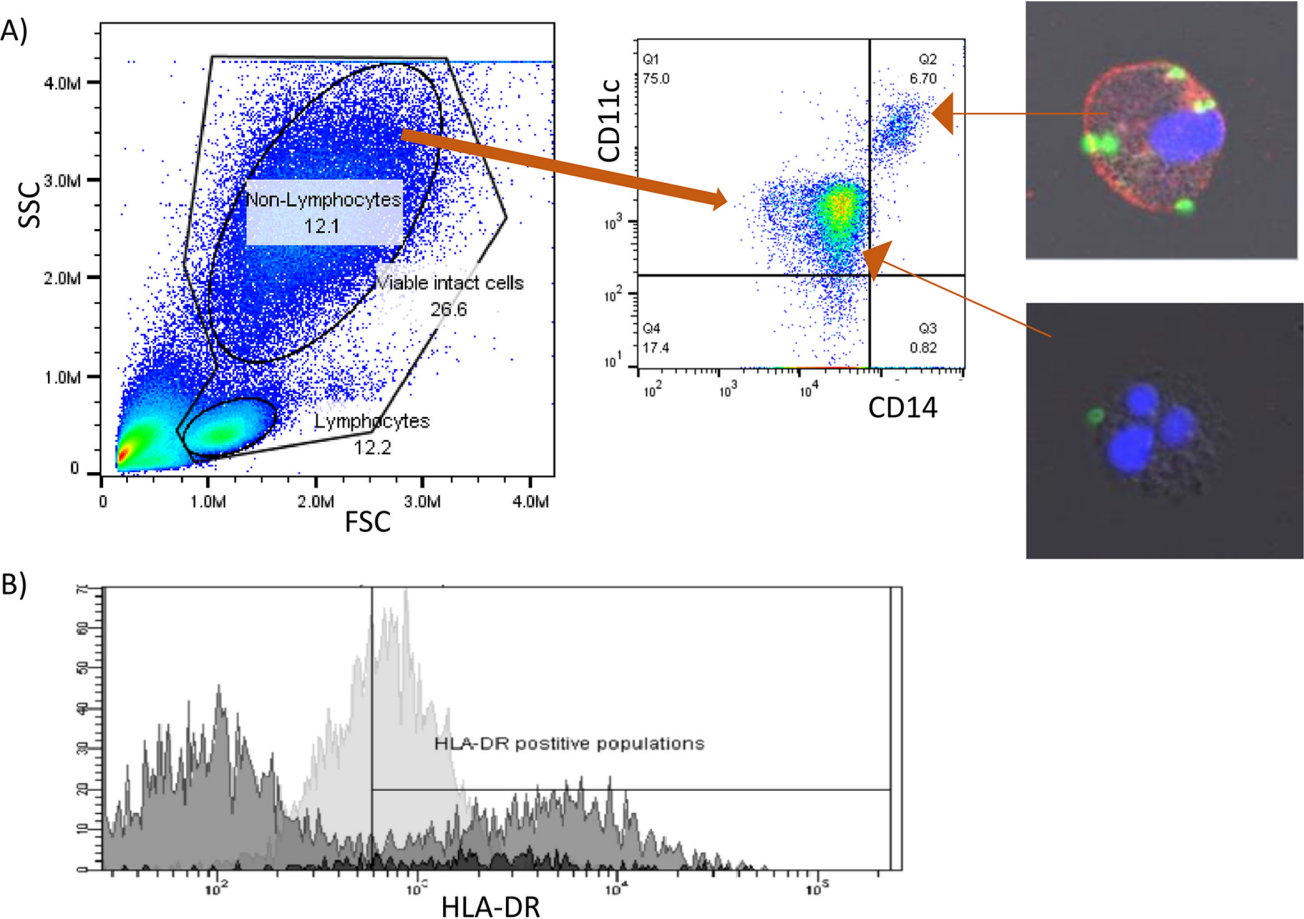
Identification of neutrophils. (A) Flow cytometry of white blood cells gated by size measured by forward scatter (FSC-A) and granularity measured by side scatter (SSC-A) showing area of interest as nonlymphocytes. Nonlymphocytes were then differentiated by staining intensity of CD11c and CD14, neutrophils were CD11c^wk^ CD14–, and monocytes were CD11c^+^, CD14^+^. The identity of monocytes was further confirmed by confocal imagery stained for CD14 (red) nuclear stain (blue), phagocytosis of B. pseudomallei stained by DSTL in-house antibody 4VIH12 (green), and the identity of neutrophils confirmed by multilobed nucleus (blue) and lack of CD14 staining and phagocytosis. (B) Flow cytometry fluorescent intensity trace showing gating for HLA-DR positive populations of neutrophils (pale gray) and monocytes (black) using lymphocyte cell staining (gray) to provide an internal control.

**FIG 2:**
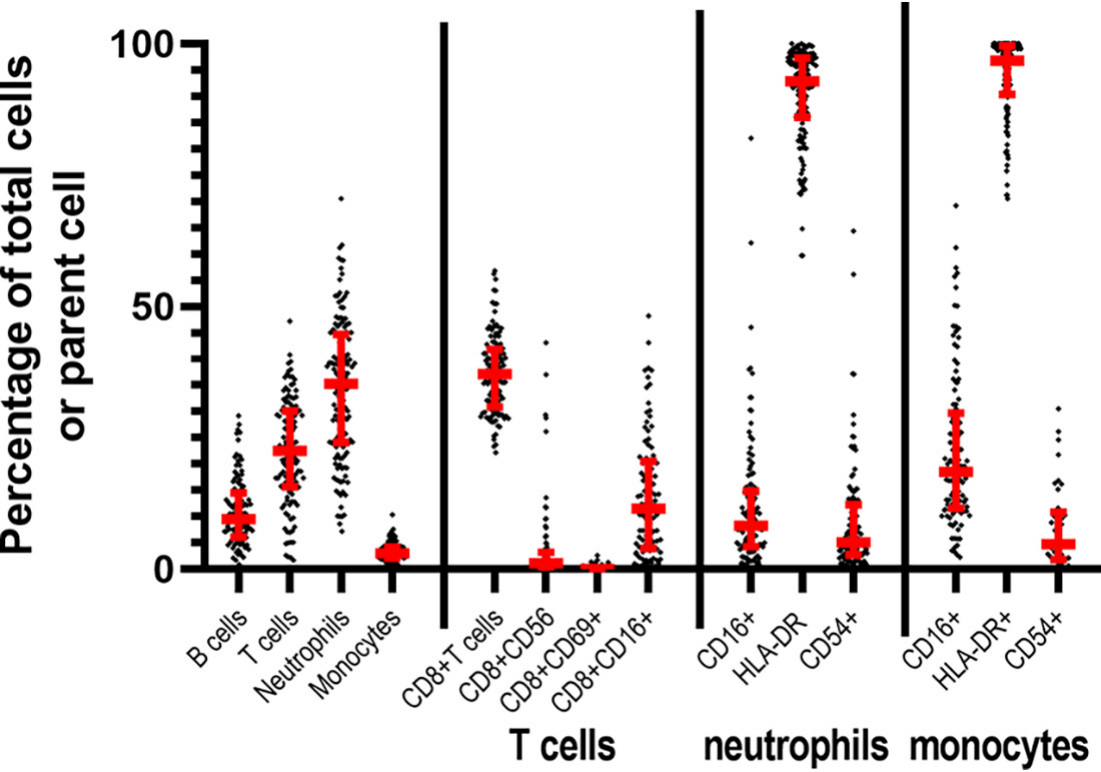
Cell phenotype and activation of white blood cells from naive marmosets. Sample size between 100 and 250 individual animals depending on marker of interest. Median and interquartile ranges are shown in red.

Over 100 naive blood samples were taken from individual animals and used to determine normal cell phenotypes and activation markers (Fig. 1 and 2). Levels of major cell types (especially in terms of the proportion of circulating neutrophils) were closer to human values than levels of peripheral blood mononuclear cells found in mice. For both monocytes and CD8 T cells, the activation markers used were expressed as expected (https://www.biolegend.com/en-us/explore-and-learn); however, naive marmoset neutrophils differed from human neutrophils. HLA-DR (MHCii) appears constitutively expressed on healthy marmoset neutrophils (sample size 250), whereas its expression is limited on human neutrophils to specific conditions or events (24). CD16, which is considered to be a defining characteristic of mature human neutrophils, was very much reduced on normal marmoset neutrophils (sample size 250) (25).

### The proportion of intact identifiable neutrophils changed during the course of disease.

**FIG 3:**
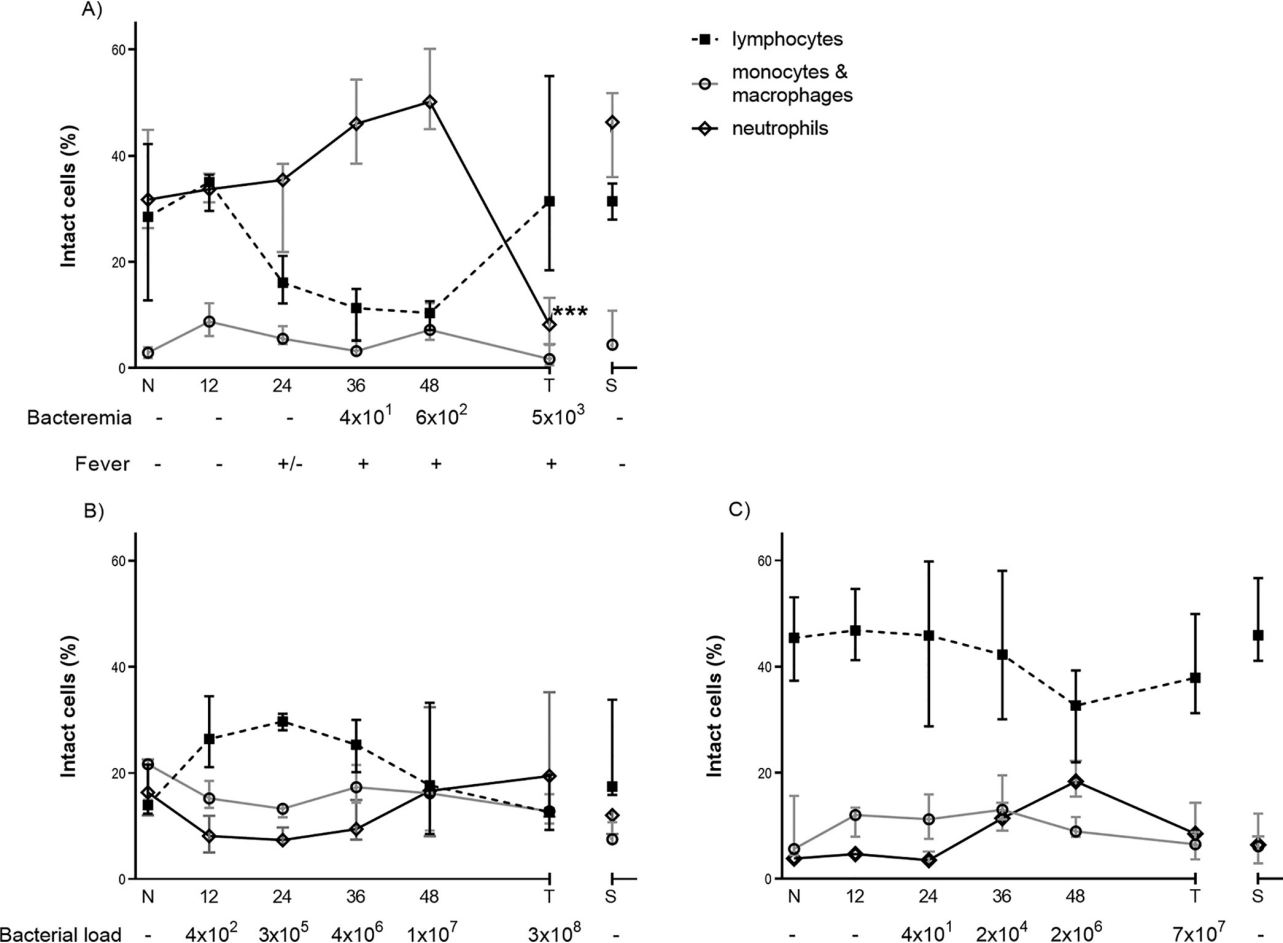
Granulocytes, macrophages, and lymphocytes from marmosets euthanized at different times following challenge with B. pseudomallei by the inhalational route. (A) Blood, (B) lung, (C) spleen. Error bars show median and interquartile ranges. N, naive animals; T, terminal disease, humane endpoint; S, survivors. Mean level of bacteremia (LOD 5cfu/mL) or bacterial load per gram of associated sample (approximately LOD 10cfu/g lung and 100cfu/g spleen) is shown below with presence (+) or absence (–) of fever. Significance difference from baseline determined by ANOVA.

The proportions of the major cell types, granulocytes, macrophages/monocytes, and lymphocytes (expressed as a percentage of total cells), together with the bacterial load, were determined in blood and from homogenates of both the lung and the spleen at 12-h time points during the disease course (Fig. 3). There was a steady rise in the neutrophil percentage in the blood during the course of disease, peaking at 48 h postchallenge. This was followed by a significant loss of these cells in the terminal samples, with levels falling below baseline levels (*P* < 0.001 by ANOVA, Fig. 3A). There was a corresponding reduction in the proportion of lymphocytes, which was predominantly caused by changes in T cells proportions, with the proportion of B cells remaining constant. In the lung, there was a decline in both the percentage of neutrophils and macrophages identifiable from the earliest time point until 48 h postinfection, when levels returned to baseline amounts. Levels of lymphocytes increased at 12 h postchallenge before declining again at 36 h postchallenge. Changes in the spleen populations were less dramatic but mirrored those observed in the blood.

### There was an immediate change in the markers expressed on the intact neutrophils in both tissue samples and blood in response to infection.

**FIG 4:**
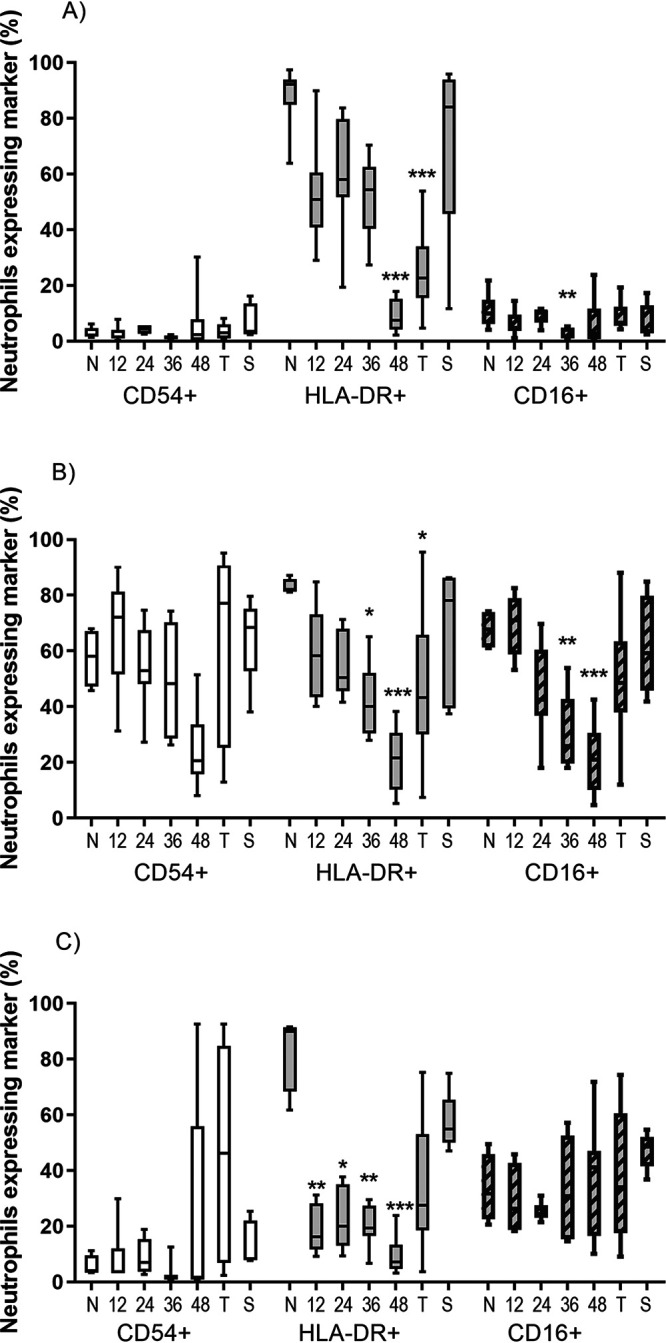
Expression of neutrophil activation markers from marmosets euthanized at different times following challenge with B. pseudomallei by the inhalational route in (A) blood, (B) lung, and (C) spleen. Data is presented as Tukey’s box and whisker plots. N, naive animals; T, terminal disease, humane endpoint; S, survivors. Significance difference form baseline (naive samples) using Dunn’s posttests is marked (***, *P* < 0.001; **, *P* < 0.01; *, *P* < 0.05).

The levels of activation or maturity markers expressed on the neutrophils changed significantly during disease. In particular, there was a reduction of the HLA-DR marker, which is highly expressed on normal functional marmoset neutrophils. The expression of this marker continued to reduce in all tissues, as the disease progressed, possibly suggesting a blocking of the binding site (Fig. 4). HLA-DR expression on neutrophils in the spleen and blood was dramatically reduced by 12 h, suggesting that there was an early systemic response to disease despite the lack of bacterial dissemination and the absence of overt clinical signs.

The decrease in neutrophil expression of HLA-DR coincided with loss of activation markers CD16 (neutrophil maturation marker) and CD54 (adhesion/migration marker) in the lung. This trend was not as apparent in either the spleen homogenates or the blood.

### T cells were activated in both lung and spleen samples at all time points postchallenge.

**FIG 5:**
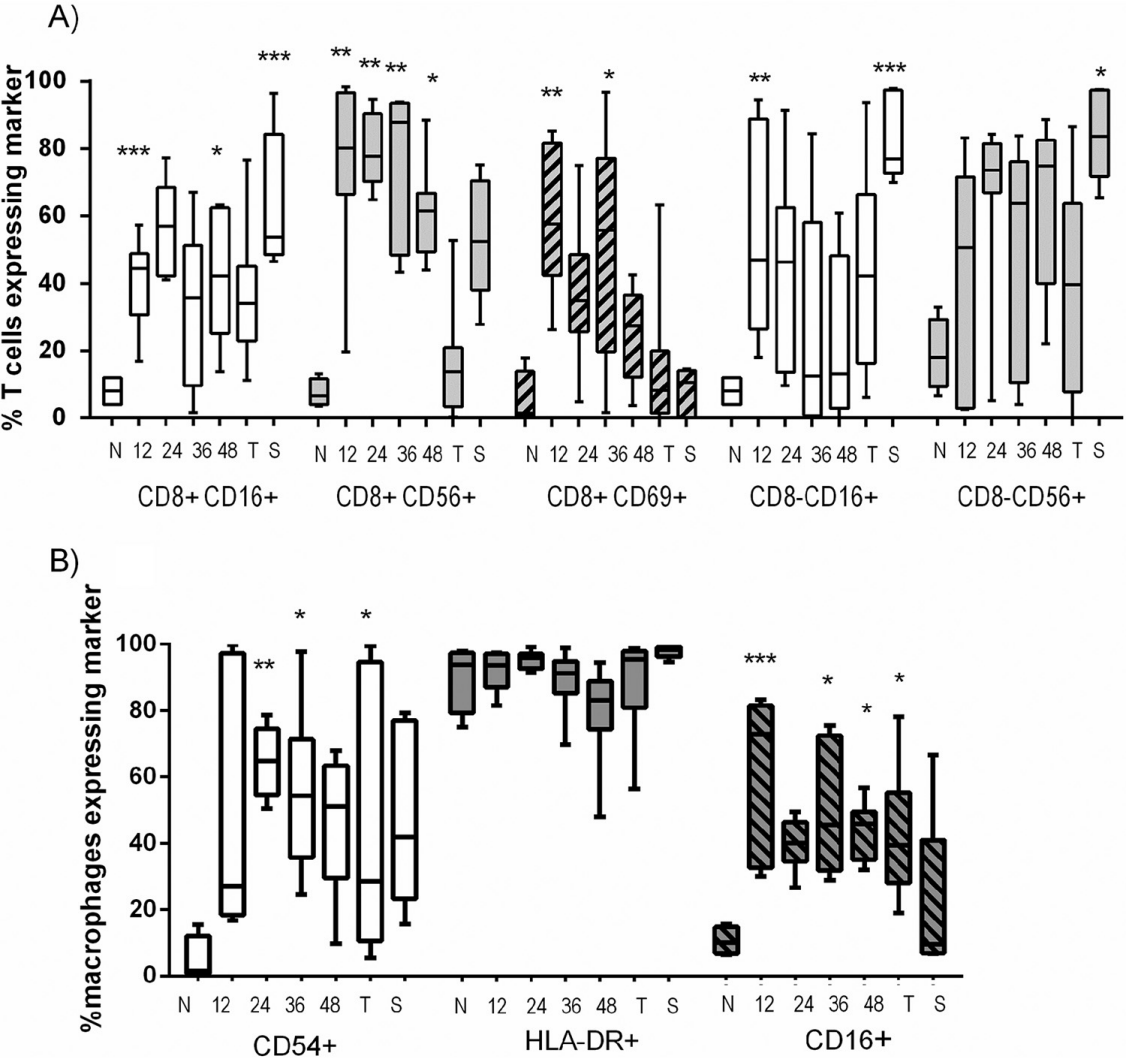
The proportions of (A) CD8^+^ and CD8– CD3^+^ T cells and (B) macrophages expressing activation markers from the lungs of marmosets euthanized at different times following challenge with B. pseudomallei by the inhalational route. Data are presented as Tukey’s box and whisker plots. N, naive animals; T, terminal disease, humane endpoint; S, survivors. Significance from naive (baseline) values is marked (***, *P* < 0.001; **, *P* < 0.01; *, *P* < 0.05).

From the earliest time point (12 h), activation of T cells was apparent and expression levels remained elevated throughout the disease time course, especially in the lungs (Fig. 5A). CD8^+^ T cells from lung homogenates were significantly upregulated for CD16, CD56, and CD69 by 12 h postinfection. There was a steady decline in the expression of these markers as the disease progressed. This was also observed for the expression of CD16 on CD8- T-cells. However, the expression of CD56 on the CD8- T-cells remained high throughout the disease progression. Many of these activation markers were also significantly upregulated on splenic T cells, at 12 h, despite bacteria only being confined to the lung at this stage of disease (data not shown). These trends were not apparent in the blood, where the expression of activation markers was more variable during disease but remained low (data not shown).

The activation status of both the CD8^+^ and CD8- T-cells remained elevated in the five animals that survived a low challenge dose of B. pseudomallei, with the exception of the early activation marker, CD69. This was more pronounced in the lungs of the three animals that had low levels of culturable *B. pseuodmallei*.

NK cells (CD3- CD56^+^) constituted a minor proportion of the cells in all samples at all time points; neither percentage nor activation (upregulation of CD16) appeared to be linked to disease even in lung samples (data not shown).

### Macrophages in the lung samples were activated from 12 h.

The proportion of cells that macrophages contributed toward the leukocyte population was initially reduced in the lung homogenates. However, the macrophages present were activated as measured by the increased expression of either CD16 or CD54 (Fig. 5B). There was also an increase in CD54 expression on spleen macrophages at the 12-h time point, which remained elevated for the whole disease course and reached significance at the humane endpoint (*P* < 0.05) (data not shown). Splenic marmoset macrophages constitutively expressed CD16. Reduction in expression of the HLA-DR marker (a recognized indicator of sepsis in humans) occurred as the disease progressed, most noticeably at 48 h postexposure (significant in the spleen and blood samples).

### There was a significant increase in all of the proinflammatory cytokines; increasing as the disease progressed.

**FIG 6:**
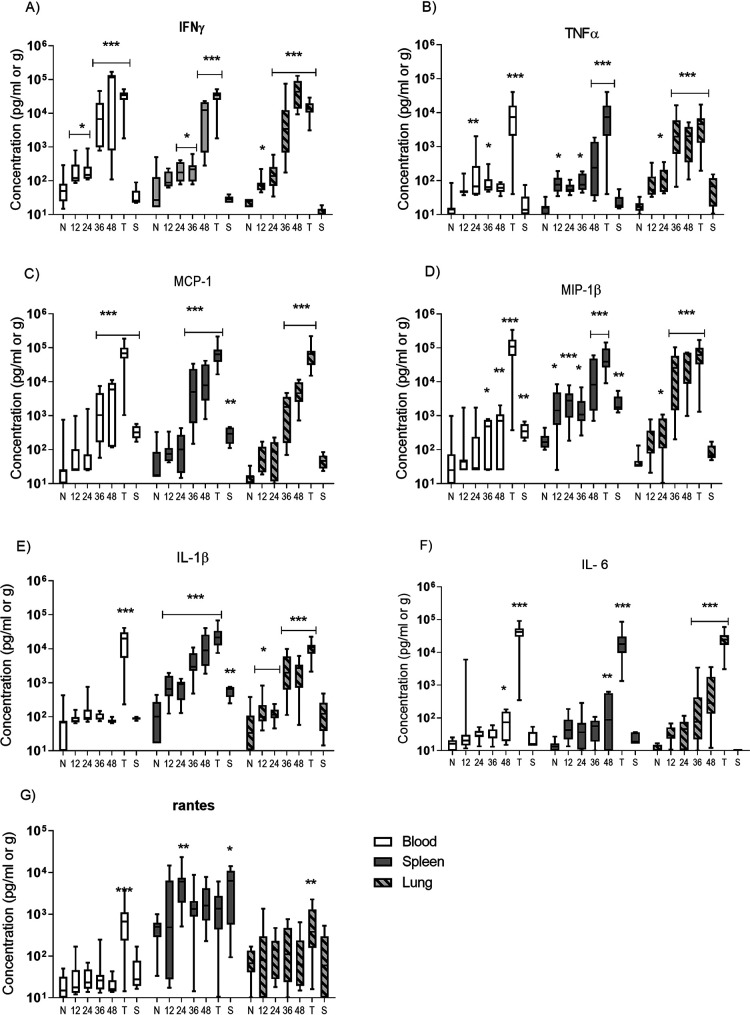
Concentration of cytokines from marmosets euthanized at different times following challenge with B. pseudomallei by the inhalational route. (A) IFNγ, (B) TNF-α, (C) MPC, (D) MIP-1β, (E) IL-1β, (F) IL-6, (G) Rantes. Tukey’s box plot, clear boxes plasma, gray boxes spleen, and striped boxes lung supernatants, N, naive samples; T, terminal samples; S, survivors. Significant difference from baseline (naive samples) using Dunn’s posttests is marked (***, *P* < 0.001; **, *P* < 0.01; *, *P* < 0.05).

Changing levels of proinflammatory cytokines (IFN-γ, TNF-α, IL-6, IL-1β) and chemokines (MCP-1, MIP-1β and RANTES) were detected in blood and tissues (Fig. 6). All the cytokines/chemokines were increasingly elevated during the course of disease in all of the samples analyzed, with the exception of RANTES. Peak concentrations were observed from the animals that were euthanized at the humane endpoint. In the plasma, all of the cytokines except RANTES were noticeably elevated as early as 12 h postinfection. IFNγ was significantly elevated at this time (*p* < 0.05), and TNF-α was significantly elevated by 24 h (*p* < 0.01). Levels of MCP-1 and MIP-1β were significant elevated at 36 h (*P* < 0.001 and 0.05, respectively). Generally, cytokine levels in the lung were more pronounced and occurred earlier than the corresponding levels observed in the spleen. In particular, levels of IFN-γ were significantly raised in the lungs at 12 h (*p* < 0.05), 12 h before the spleen, and also for IL-6, 36 h in the lung compared to 48 h in the spleen.

Generally, cytokine levels in animals that survived challenge with a low dose of B. pseudomallei and remained afebrile (labeled S, Fig. 6) were similar to naive values. However, levels of some cytokines remain significantly elevated, namely, MPC-1 in the spleen, and IL-1β and MIP-1β in both spleen and plasma samples.

### Changes in the levels of HLA-DR expression on blood neutrophils provided an early indicator of acute disease.

**FIG 7:**
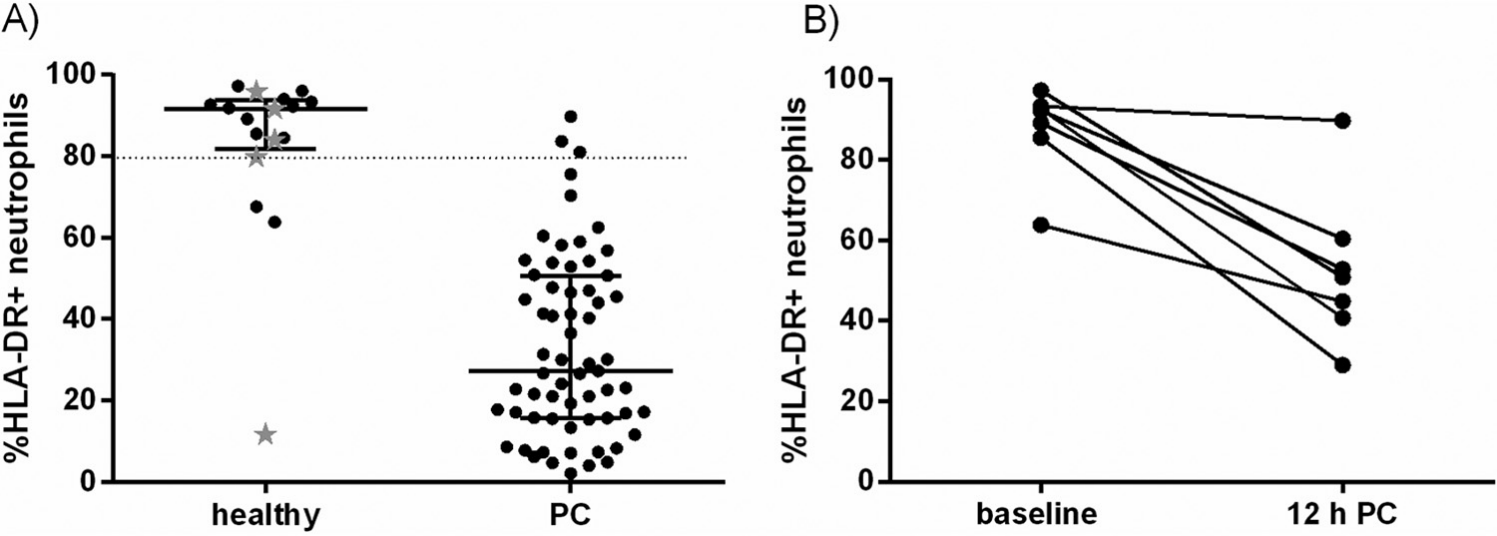
Dynamics in expression of HLA-DR on neutrophils in blood samples from marmosets taken before challenge (baseline/healthy), postchallenge (PC), and 12 h PC with B. pseudomallei by the inhalational route. (A) Healthy includes baseline and low challenge animals (gray stars), (B) paired samples.

High (positive) HLA-DR expression on blood neutrophils was determined by using lymphocyte staining as an internal control (Fig. 1). Healthy animals had a median level of 92% positive, significantly more than the postchallenge median of 27% (Fig. 7A; *P* < 0.0001 Student's *t* test). Assessing all the blood samples included in this study, it was determined that a staining level of greater than 80% positive could be used to distinguish between “healthy” and “sick” animals. This staining level would have predicted that 14/17 (82%) healthy animals were healthy (prechallenge and low-challenge survivors) and that 60/63 (95%) of the postchallenge sick animals (including those euthanized at the humane endpoint) were sick. When pre- and postchallenge blood sample levels were compared, neutrophil HLA-DR was significantly reduced at 12 h postchallenge when compared to matched baseline levels (*P* = 0.004 by paired *t* test). In this case, 6/7 (86%) animals had a greater than 10% reduction in expression (Fig. 7B). However, there was no direct relationship between neutrophil HLA-DR expression levels and bacterial counts for each sample.

### Key characteristics from single blood samples were able to discriminate between most healthy and apparently healthy 12–24 h postchallenge animals and all animals 36–48 h postchallenge.

**FIG 8:**
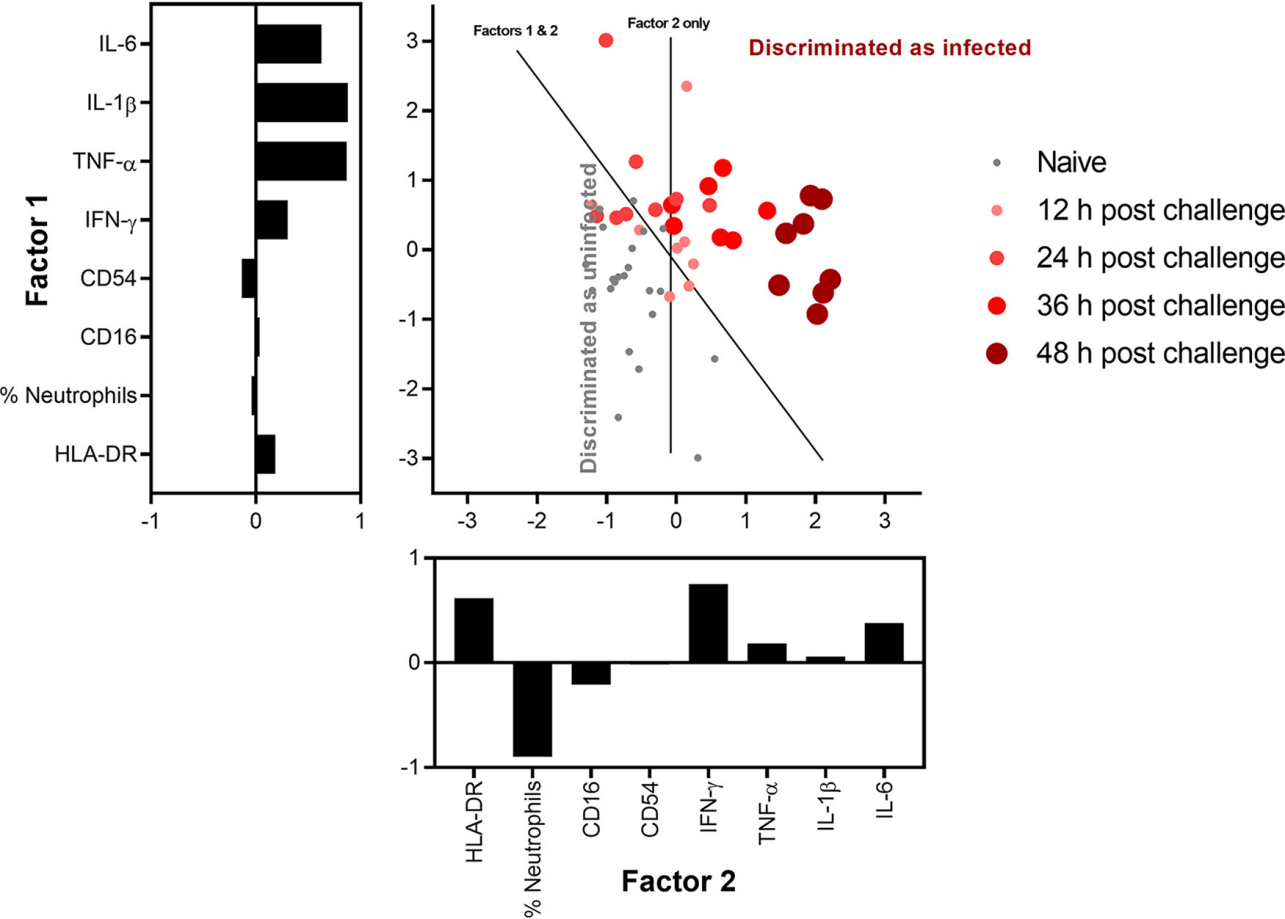
Principal-component analysis of data from blood samples taken from uninfected animals and animals postchallenge with B. pseudomallei. Metrics include 4 activation states of neutrophils and 4 inflammatory cytokines. The scale of the contribution of the 8 metrics applied to each component (bar charts) is shown as rotated coefficients. The data from each subject where the coefficient has been applied are shown as a scatter graph. Two additional discriminant analyses were run to differentiate between infected and uninfected animals. One discriminant analysis used only factor 2 and the other used both factors. The thresholds of where these analyses discriminate between the two conditions are represented by lines marked on the plot.

Principal component analysis (PCA) was performed to evaluate whether the HLA-DR neutrophil expression combined with other data could be applied to individual blood samples to discriminate between infected and uninfected animals and to determine the stage of infection. The analysis used eight variables: four variables relating to the proportions of neutrophils and their activation HLA-DR, CD16, and CD54 expressions, and a further four variables consisting of key inflammatory cytokines: IFN-γ, TNF-α, IL-1β, and IL-6. A total of 54 blood samples were included in the analysis, which had data for all metrics. These included blood samples taken from healthy animals (*n* = 22), or from animals at 12, 24, 36, or 48 h postchallenge. Approximately 75% of the discriminating data used was comprised of 3 principal components. For each animal, the score for these three factors were calculated (factors 1 and 2, Fig. 8). The first component accounted for aspects of variation between animals that did not relate to infection status, mainly comprised of the proportion neutrophils and cytokine release; the second component accounted for variation between animals that related to infection status, mainly composed of a proportion of the expression of HLA-DR and release of IFN-γ. Component factor 3 identified a difference that existed for two animals (one infected, one not, data not shown) that mainly comprised cell activation.

PCA using just factor 2 or using factor 1 and 2 combined was able to identify animals that had been infected for 36 and 48 h postchallenge with 100% sensitivity; however, it could not differentiate between the naive animals and the 12 and 24 h postchallenge animals with any accuracy (Table 1). Across all groups, the single component discriminant model had a specificity of 91% and sensitivity of 72%, while the two-component discriminant model had a specificity of 91% and sensitivity of 78% (when considering all times after challenge). These factors relied most heavily on the proportion of neutrophils, expression of neutrophil HLA-DR, and presence of IFN- γ; these 3 factors assessed across paired blood samples provided highly reliable biomarkers of severe melioidosis, measurable before onset of clinical signs.

**TABLE 1:** The capability of a discriminant analysis model to differentiate between animals in different disease states using principal components of immune analysis of blood^*a*^

Factors used in analysis	Actual group	Predicted group		Proportion success	Specificity^*b*^	Sensitivity
		Naïve	Infected			
Factor 2 only	Naïve	20	2	20/22	91% (72, 98)	-
	12 h postchallenge	3	5	5/8	-	63% (31, 86)
	24 h postchallenge	6	2	2/8	-	25% (4, 59)
	36 h postchallenge	0	8	8/8	-	100% (68, 100)
	48 h postchallenge	0	8	8/8	-	100% (68, 100)
	Overall		Infected	23/32	91% (72, 98)	72% (55, 84)

Factors 1 & 2	Naïve	20	2	20/22	91% (72, 98)	-
	12 h postchallenge	4	4	4/8	-	50% (22, 78)
	24 h postchallenge	3	5	5/8	-	63% (31, 86)
	36 h postchallenge	0	8	8/8	-	100% (68, 100)
	48 h postchallenge	0	8	8/8	-	100% (68, 100)
	Overall		Infected	25/32	91% (72, 98)	78% (61, 89)

a These were based on four activation states of neutrophils and four inflammatory cytokines. Percentages are given to 2 significant figures. The 95% confidence intervals for the percentages are given (using the Wilson-Brown method).

b Dashes indicate where specificity/sensitivity calculations were not performed due to the uniform nature of the data.

## DISCUSSION

This is the first report tracking the innate immune response of NHPs to acute fatal melioidosis by both cellular and cytokine responses. There are clear signs of upregulation in cell activity from the earliest time point assessed, with the most dramatic changes occurring first in the lung, the organ of insult, spreading through the body with bacterial dissemination.

Neutrophils showed the biggest response to disease. This was expected given that neutrophils are considered to be the first responders and their significance in early melioidosis has been previously reported (17, 19). In contrast to the mouse model where there was an influx of neutrophils into the lung, (19), a reduction/depletion in the proportion of neutrophils was observed in marmosets from 12 h postchallenge. As the cell typing was proportional, it was difficult to determine if this change was due to an influx of lymphocytes rather than death or degeneration of the neutrophils by bactericidal processes such as generation of neutrophil nets (26). Neutrophil proportions within the lung samples started to recover at 36 h; by 48 h, lung, spleen, and blood all had increased proportions of neutrophils.

Recruitment of functional, activated neutrophils into the lung in a mouse model of melioidosis is absolutely critical in controlling an acute aerosol infection (17). The susceptibility of the marmoset to B. pseudomallei may be related to the observed decline in neutrophils, provided this is not a function of other populations increasing more rapidly. It is also possible that the marmosets recruited neutrophils immediately, but they were heavily infected and too degenerate to be detected by flow cytometry. This theory is supported by the blood analysis data and lung histology data reported elsewhere (22). Further neutrophil recruitment later in the disease process was too late, and these cells were undeveloped and ineffective.

The pattern of a peak in neutrophils in the blood 1 to 2 days postchallenge has been reported in other NHP models of melioidosis, with a rapid decline thereafter being linked to poor prognosis (14). The presence of either excessively high or excessively low counts of neutrophils in blood from human patients has been linked with poor outcomes (21), and the prevalence of melioidosis is increased in conditions associated with poor neutrophil function (e.g., diabetes) (18, 27).

The clearest indication of neutrophil involvement was the reduction in HLA-DR expression, reduced on all neutrophils regardless of where they were isolated. Expression of the HLA-DR marker, which is a member of the Major Histocompatibility class II molecules, is not normally associated with human neutrophils (24, 25) but appears to be constitutively expressed on normal functional marmoset neutrophils. Reduction of this marker on human macrophages is linked to serve infection and sepsis, and is taken as an indicator of immune suppression or immune paralysis (28–30). It has also recently been observed in fatal human cases of melioidosis (31). In this study, there was some reduction in the expression of HLA-DR on macrophages, but the change in expression on neutrophils was highly statistically significant and linked to disease. Blood neutrophils had significantly reduced expression of HLA-DR at all time points postchallenge. There was a significant reduction in HLA-DR expression on neutrophils within the spleen at 12 h, which was 12 h before the onset of overt signs of disease, specifically fever. This change in neutrophil expression suggests that there was a systemic response to disease despite bacteria at this time being localized to the lung. At 12 h postchallenge, six out of seven animals had at least a 10% reduction in HLA-DR expression on blood neutrophils compared to baseline levels. This may be a useful biomarker of disease in this model and could be used as a trigger to treat. In a study on critical care patients, a 5% reduction in blood monocyte expression between paired samples has been suggested as a trigger to treat (29). This aligns with the requirement for well characterized animal models consistent with human disease, for licensure of medical countermeasures under the FDA Animal Rule (23). This could be further expanded as composite analysis, using four neutrophil characteristics and four cytokine levels. This would allow single blood samples to be used to identify infected animals before clinical signs develop. The use of the neutrophil HLA-DR expression alone to assess the animal’s health is more powerful when directly compared to each animal’s baseline values, as the 2 healthy animals with low baseline values had further reductions postchallenge.

Another neutrophil marker, CD16, is considered to be a maturity marker for functional neutrophils (32). The loss of this marker from the neutrophils in the lung samples at the later time points could be a measure of a left shift, which indicates that functional mature neutrophils are being destroyed faster than they can be replaced. Determination of the left shift on human neutrophils had long been used as a diagnostic marker of serve or long-term infection (33). That the expression of HLA-DR was independent of CD16 on the spleen neutrophils might indicate HLA-DR is a functional change rather than a maturity marker.

There was no obvious recruitment of macrophages to the lungs, but the macrophages that were present were appropriately activated based on CD16 expression. In mouse models, macrophages are essential and depletion experiments resulted in increased disease severity (34). The acute infection model in BALB/c mice is associated with neutrophil influx, whereas the more resistant C57BL/6 mouse model has a marked influx of macrophages (35). However, *in vitro* studies using human peripheral blood mononuclear cells (PBMC) have shown that monocytes support replication of B. pseudomallei (36). Poor IFN-γ production and subsequent monocyte activation have been suggested as the cause of increased susceptibility of diabetics to melioidosis (37).

The cytokines TNF-α, IL-1β, and IL-6 are known to be predictors of poor outcome in human disease, in particular increased levels of IL-6 (38), and these were noticeably elevated in the marmoset plasma samples early in the disease. These cytokines were significantly elevated in spleen homogenates as well, even at early time points, again suggesting a systemic response to disease. IFN-γ was the first cytokine to be significantly elevated and was produced in excessive quantities. IFN-γ is essential for limiting bacterial growth (5), but there are many studies suggesting that overproduction of IFN-γ causes the BALB/c mouse to be more susceptible than the C57BL/6 (17). The source of this cytokine has been shown in mice to be both NK and CD8 T cells (6, 39, 40). In the marmoset, there was a significant increase in IFN-γ in the lung from 12 h, the source of which is most likely to be activated T cells. Conversely activation of both CD4 and CD8 T cells, in terms of production of IFN-γ, in blood samples from human melioidosis has been linked to survival, and a reduced T cell response in diabetics is possibly linked to their poorer outcome (21).

Although high levels are linked to poor outcomes, the controlled production of TNF-α, IL-6, MCP-1, and IFN-γ in the C57BL/6 mouse model is essential to control of disease (17). In the marmoset model, these cytokines were significantly increased during disease; however, the increase in IFN-γ was much greater than for TNF-α or IL-6, and may represent the uncontrolled production seen in the BALB/c mouse acute model (9). In rhesus macaques and African green monkeys, comparable levels of IL-6, IL-1β, and TNF-α to the marmoset were detected. However, much lower levels of IFN-γ were observed, and a significantly longer time to death (14). Human PBMCs *in vitro* produce large quantities of these proinflammatory cytokines in response to a killed related bacteria, B. mallei, with similar responses in three different NHPs toward B. mallei or B. pseudomallei (41). In a recent study (7), high levels of IFN-γ as well as substantial increases in IL-6, IL-1β, MIG, and IL-10 were linked to the poor outcome of BALB/c compared to C57BL/6, which produced less IFN-γ and also almost no detectable increase in these other cytokines.

Other features of the marmoset model, including high mortality with high fever, pulmonary distress, and hepatic dysfunction, as well as hepatosplenomegaly, hepatic necrosis, and lymphadenopathy (22), are also features of acute human melioidosis (42–46).

Three out of five of the animals that survived low challenge doses had viable bacteria in their lungs, suggesting that they had received an infectious but not lethal dose. These animals may have eventually succumbed to disease, although the increased levels of T cell activity detected could be linked to a developing adaptive immunity. Lower than expected neutrophil HLA-DR expression was observed in the blood and spleen, possibly indicating an ongoing disease, even though these animals did not display clinical signs. Interestingly, principal-component analysis (PCA) placed all but one of these animals in the healthy group; that individual animal had a greater than 20% reduction in HLA-DR pre- to postplasma samples.

Despite the widespread endemic nature of melioidosis in some counties, there are a limited number of identified inhalational cases, which limits the amount of patient data that can be used to define and evaluate the disease parameters. This study provides a detailed immunological profile in the marmoset of acute disease with poor prognosis, thus providing a range of features against which to evaluate the efficacy of medical countermeasures.

## MATERIALS AND METHODS

The marmosets used to provide immunological data are from a number of different studies, aimed at developing and characterizing the inhalational model and which have been reported elsewhere (9, 22). Full experimental details are given in these publications. In order to have large enough group sizes to allow meaningful analysis, this study combines data from infection studies using 2 different strains of B. pseudomallei, determined to produce similar pathology (22).

### Bacterial strain and culture.

B. pseudomallei strains, K96243 (laboratory passaged strain) or recent clinical isolate HPUB10303a (supplied by Biomedical Research and Development Authority [BARDA] from Battelle Biomedical Research Centre or Public Health England, United Kingdom), were prepared as described previously (22).

### Marmosets.

Healthy sexually mature common marmosets (*C. jacchus*) were obtained from the DSTL Porton Down breeding colony and housed in vasectomized male and female pairs. They were between 18 months and 5 years old and weighed between 320g and 500g. All animals were allowed free access to food and water as well as environmental enrichment. All animal studies were carried out in accordance with the UK Animals (Scientific Procedures) Act of 1986 and the Codes of Practice for the Housing and Care of Animals used in Scientific Procedures 1989.

Animals were challenged via the inhalational route using a contained Henderson apparatus controlled by the AeroMP (Aerosol Management Platform) aerosol system (Biaera Technologies LLC, Hagerstown, MD, United States) with either B. pseudomallei K96243 or HPUB10303a. Animals were euthanized at various time points postchallenge or when they had reached a humane endpoint. Two weeks prior to the challenge, animals had blood collected to determine baseline immunological parameters.

Forty animals were infected by the aerosol route with a retained dose of 40.8 CFU (1–201), which gave an average time to death (humane endpoint) of 71.6 h (53.4–81.7). Of the 16 marmosets that received less than 10 CFU, 5 survived 7 days (or more) without a measured temperature increase and 2 of these animals had no culturable B. pseudomallei bacteria in organs postmortem. All other animals had fever at 24 h and reached a fever plateau by 36 h.

Samples were also available from a further 32 animals, which were infected with a retained dose of 47.9 CFU (16–123) and euthanized in groups of 8 at 4 time points postinfection: 12 h before onset of fever or signs; 24 h low grade fever apparent in most animals; 36 h fever in all animals, most having high grade fever; and 48 h high fever and other mild clinical signs. Bacterial counts were determined in key organs at each time point.

Additionally, three animals of similar age were used to provide naive samples.

### Flow cytometry on leukocyte populations.

Tissue samples were homogenized to provide single cell suspensions (15). Red blood cells were lysed (red cell lysis buffer BD Bioscienecs), and the mixed leukocyte population was washed and stained with the following combinations of the mouse antihuman fluorescent antibody stains: for lymphocytes CD3 (SP34-2), CD8 (LT8), CD56 (B159), CD69 (FN50), CD20 (Bly1), CD16 (3G8); for monocytes/macrophages and neutrophils CD11c (SHCL3), CD14 (M5E2), CD16 (3G8), CD54 (HCD54), CD163 (GHI/61), and HLA-DR (L243) (BD Bioscience, BioLegend, AbD Serotec). All samples were fixed in 4% paraformaldehyde for 48 h at 4°C and analyzed by flow cytometry (FACScanto II BD, using data package DIVA) within 72 h of staining. Basic cell types (lymphocytes, monocytes, and granulocytes) were determined by forward and side scatter, and cellular debris differentiated from intact cells by nuclear staining (Fig. 1). Neutrophils were differentiated from monocytes by the intensity of CD11c and CD14 staining (12), and validation of this was provided by confocal microscopy of nuclear morphology and *ex vivo* phagocytosis of B. pseudomallei visualized by DSTL in-house anti B. pseudomallei antibody 4VIH12. HLA-DR staining on the lymphocyte population was used to provide an internal control for determining positive populations for neutrophils and monocytes (Fig. 1B). Cell populations were expressed as proportions of viable intact cells and activation expression as a proportion of the parent cell type.

Levels of cytokines and chemokines were also quantified in the plasma or tissue homogenates using the human flexset for IL-1β, IL-6, MIP-1β, MCP-1, and RANTES (BD cytokine flex beads) and for TNF-α and IFN-γ (marmoset specific reagents from U-CyTech Biosciences and Mabtech AB, conjugated by BBI Detection, Ltd). Samples were processed according to manufacturer’s instructions and then fixed in 4% paraformaldehyde for 48 h at 4°C before being analyzed by flow cytometry (FACScanto II BD); concentrations were calculated per mL of blood or g of homogenized tissue (20).

### Statistics.

Statistical analysis, Kruskal Wallis tests, and ANOVAs were performed using GraphPad PRISM V6.0. The statistical package SPSS V21.0 was used for principal-component analysis. The varimax rotation method was used for this method. Cytokine data were transformed to the logarithm of 10. Factor scores for each animal were estimated by the regression method. Subsequent discriminant analyses were run on the factor scores.
